# Bacterial Microcompartment-Dependent 1,2-Propanediol Utilization of *Propionibacterium freudenreichii*

**DOI:** 10.3389/fmicb.2021.679827

**Published:** 2021-05-12

**Authors:** Alexander Dank, Zhe Zeng, Sjef Boeren, Richard A. Notebaart, Eddy J. Smid, Tjakko Abee

**Affiliations:** ^1^Food Microbiology, Wageningen University and Research, Wageningen, Netherlands; ^2^Laboratory of Biochemistry, Wageningen University and Research, Wageningen, Netherlands

**Keywords:** bacterial microcompartments, *Propionibacterium freudenreichii*, vitamin B_12_, metabolosome, propanediol utilization cluster, probiotic, intestine

## Abstract

Bacterial microcompartments (BMCs) are proteinaceous prokaryotic organelles that enable the utilization of substrates such as 1,2-propanediol and ethanolamine. BMCs are mostly linked to the survival of particular pathogenic bacteria by providing a growth advantage through utilization of 1,2-propanediol and ethanolamine which are abundantly present in the human gut. Although a 1,2-propanediol utilization cluster was found in the probiotic bacterium *Propionibacterium freudenreichii*, BMC-mediated metabolism of 1,2-propanediol has not been demonstrated experimentally in *P. freudenreichii*. In this study we show that *P. freudenreichii* DSM 20271 metabolizes 1,2-propanediol in anaerobic conditions to propionate and 1-propanol. Furthermore, 1,2-propanediol induced the formation of BMCs, which were visualized by transmission electron microscopy and resembled BMCs found in other bacteria. Proteomic analysis of 1,2-propanediol grown cells compared to L-lactate grown cells showed significant upregulation of proteins involved in propanediol-utilization (*pdu*-cluster), DNA repair mechanisms and BMC shell proteins while proteins involved in oxidative phosphorylation were down-regulated. 1,2-Propanediol utilizing cells actively produced vitamin B_12_ (cobalamin) in similar amounts as cells growing on L-lactate. The ability to metabolize 1,2-propanediol may have implications for human gut colonization and modulation, and can potentially aid in delivering propionate and vitamin B_12_ *in situ*.

## Introduction

*Propionibacterium freudenreichii* is a Gram-positive, non-spore forming bacterium which has been linked to several potential health promoting effects, such as reducing intestinal inflammation, immunomodulation, modulation of intestinal motility and absorption, reduction of pathogen adhesion and enhancement of bifidobacteria [reviewed by Cousin et al. (2011)]. *P. freudenreichii* is able to cope with a large variety of stresses (oxidative, bile salt, and temperature; Falentin et al., 2010) and consequently has good survival capabilities in the upper intestinal tract (Huang and Adams, 2004). Survival of *P. freudenreichii* in the intestinal environment is supported by expression of pathways involved in the metabolism of substrates present in the intestinal environment, such as propanediol (Saraoui et al., 2013).

1,2-propanediol is a major end product from anaerobic degradation of rhamnose or fucose by the human intestinal microbiota and serves as an important carbon source for 1,2-propanediol utilizing bacteria, which can metabolize 1,2-propanediol into propionate, generating ATP, and into 1-propanol for maintaining redox balance. The metabolism of 1,2-propanediol produces the toxic intermediate propionaldehyde (Sampson and Bobik, 2008). Some bacteria can protect themselves from toxic intermediates by encapsulating the enzymatic processes in self-assembling proteinaceous organelles called bacterial microcompartments (BMCs; Axen et al., 2014). BMCs are typically about 40–200 nm in diameter and are made of three types of shell proteins: hexamers, pseudohexamers, and pentamers (Kerfeld et al., 2018). Based on the highly conserved domain of shell proteins, Axen et al. (2014) predicted the presence of these organelles in 23 different bacterial phyla, including *Actinobacteria* which includes the species *P. freudenreichii*. However, no experimental evidence of BMC mediated utilization of 1,2-propanediol has been shown in *P. freudenreichii* and its role as carbon source is yet to be elucidated.

Bacterial microcompartment-mediated catabolism of substrates involving a toxic aldehyde intermediate is driven by three core enzymes: aldehyde dehydrogenase, alcohol dehydrogenase, and phosphotransacylase (Axen et al., 2014). The signature enzyme in the propanediol utilization (*pdu*) pathway is propanediol dehydratase, a vitamin B_12_-dependent enzyme catalyzing the reaction of 1,2-propanediol to propionaldehyde (Cheng et al., 2011; Axen et al., 2014). Vitamin B_12_ is produced in high quantities by *P. freudenreichii* (Burgess et al., 2009) and it also requires vitamin B_12_ as a cofactor for a key enzyme in its characteristic Wood–Werkman cycle. It is suggested that *Actinobacteria* such as *P. freudenreichii* obtained the *pdu* cluster through horizontal gene transfer from *Clostridiales* (Ravcheev et al., 2019), in which acquisition may have been supported by the ability of *P. freudenreichii* to produce vitamin B_12_ *de-novo* (Roessner et al., 2002). BMCs are also found in some pathogenic bacteria, such as *Salmonella enterica, Enterococcus faecalis, Listeria monocytogenes*, pathogenic *Escherichia coli*, and *Clostridium perfringens* (Kerfeld et al., 2018). BMC-mediated utilization of 1,2-propanediol increases competitive fitness of pathogens in the gut and consequently has been linked to virulence (Jakobson and Tullman-Ercek, 2016). However, symbiotic relationships depending on 1,2-propanediol metabolism have also been shown for beneficial *Lactobacillus reuteri* and *Bifidobacterium breve* (Cheng et al., 2020). The ability to degrade 1,2-propanediol may have similar implications for the bifidogenic capacity reported for *P. freudenreichii* (Kaneko et al., 1994) and consequences for gut modulation and competition with pathogenic bacteria. Furthermore, the active production of vitamin B_12_ during metabolism of 1,2-propanediol has not been studied yet in *P. freudenreichii*.

In this study we present evidence for BMC-mediated anaerobic growth of *P. freudenreichii* on 1,2-propanediol, evidenced by substrate utilization, propionate and 1-propanol production, and vitamin B_12_ synthesis. Using transmission electron microscopy (TEM) and proteomics, we confirmed the presence of BMCs, Pdu BMC shell proteins and enzymes in *pdu*-induced *P. freudenreichii*.

## Materials and Methods

### Strains, Culture Conditions, and Growth Measurement

*Propionibacterium freudenreichii* DSM 20271 was grown anaerobically (Anoxomat modified atmosphere, MART; 10% CO_2_, 5% H_2_, and 85% N_2_) at 30°C in 50 mL tubes containing 40 mL complex media containing per liter: 10 g tryptone, 5 g yeast extract, and 5 g KH_2_PO_4_ supplemented with 100 mM L-lactate or 1,2-propanediol. All media was set at pH 7.0 by addition of 5 M NaOH and was filter sterilized through 0.2 μm filters into sterile flasks. OD_600_ measurements and extracellular metabolite samples were taken daily for a time period of 7 days.

### Analysis of Extracellular Metabolites Using High Performance Liquid Chromatography

Culture samples were taken at various time intervals and were analyzed for extracellular metabolites by high performance liquid chromatography. 1 mL culture was centrifuged at 17,000 × *g* for 1 min and the supernatant was collected. 0.5 mL supernatant was treated with 0.25 mL Carrez A and 0.25 mL Carrez B, vortexed and centrifuged at 17,000 × *g* for 2 min. 200 μL supernatant was stored in HPLC vials at −20°C upon analysis. HPLC was performed as described by Zeng et al. (2019). Quantification was performed by addition of a standard curve containing L-lactate, acetate, propionate, 1,2-propanediol, and 1-propanol.

### Transmission Electron Microscopy

*Propionibacterium freudenreichii* cultures were grown anaerobically at 30°C in 100 mM L-lactate or 1,2-propanediol containing media. Samples were collected after 6 days of incubation (early stationary phase for 1,2-propanediol-grown cells). About 10 μg of dry cell biomass was fixed for 2 h in 2.5% (v/v) glutaraldehyde in 0.1 M sodium cacodylate buffer (pH 7.2). After rinsing in the same buffer, a post-fixation was done in 1% (w/v) OsO_4_ for 1 h at room temperature. The samples were dehydrated using ethanol. The dehydrated cell pellets were then embedded in resin (Spurr HM20) for 10 h at 70°C. Thin sections (<100 nm) of polymerized resin samples were obtained with microtomes. After staining with 2% (w/v) aqueous uranyl acetate, the samples were analyzed with a Jeol 1400 plus TEM with 120 kV setting as described by Zeng et al. (2019).

### Vitamin B_12_ Quantification

After 7 days of incubation the vitamin B_12_ (cobalamin) concentration was determined using a microbiological assay (Vitafast vitamin B_12_ kit, R-biopharm) for *P. freudenreichii* grown in 66 mM L-lactate and 49 mM 1,2-propanediol medium. Briefly, 1 mL of culture was disrupted by bead beating (lysing matrix B, mp-bio) 2× 1 min at 4.5 m/s with 1 min on ice in between. Samples were centrifuged and diluted with water to appropriate concentrations for the test kit and were heat-extracted for 30 min at 95°C. The samples were cooled to room temperature and vitamin B_12_ detection was performed according to the manufacturer protocol.

### Proteomics

*Propionibacterium freudenreichii* cells were cultured in media supplemented with 100 mM L-lactate or 100 mM 1,2-propanediol for 7 days. Cell pellets were harvested by centrifugation of 1 mL of sample at 17,000 × *g* for 1 min in table top centrifuges and cell pellets were frozen at -80 degrees. Samples were washed twice with 100 mM Tris (pH 8) and resuspended in 100 μl 100 mM Tris. Samples were lysed by sonication for 45 s twice while cooling 1 min on ice. Protein content was determined using Pierce Coomassie protein assay and samples were diluted to 1 μg/μl using Tris–HCl pH 8. Samples were prepared according to the filter assisted sample preparation protocol (FASP; Wiśniewski et al., 2009) with the following steps: reduction with 15 mM dithiothreitol, alkylation with 20 mM acrylamide, and digestion with sequencing grade trypsin overnight. Each prepared peptide sample was analyzed by injecting (18 μl) into a nanoLC-MS/MS (Thermo nLC1000 connected to a LTQ-Orbitrap XL) as described previously (Lu et al., 2011). LCMS data with all MS/MS spectra were analyzed with the MaxQuant quantitative proteomics software package (Cox et al., 2014) as described before (Smaczniak et al., 2012; Wendrich et al., 2017).

A protein database with the protein sequence of *P. freudenreichii* DSM 20271 (ID:UP000032238) was downloaded from UniProt. Filtering and further bioinformatics and statistical analysis of the MaxQuant ProteinGroups file were performed with Perseus (Tyanova et al., 2016). Reverse hits and contaminants were filtered out. Protein groups were filtered to contain minimally two peptides for protein identification of which at least one is unique and at least one is unmodified. Also, each group required three valid values in at least one of the two experimental groups. A volcano plot was prepared based on the Student’s *t*-test difference between samples. Volcano plots were produced in Rstudio using EnhancedVolcano (Blighe, 2018). Proteins were considered to be significantly different amongst sample if *p* < 0.05 and 4-fold change difference was detected. KEGG gene set enrichment analysis was performed using Clusterprofiler (Yu et al., 2012) and 2-fold change difference amongst proteins. The mass spectrometry proteomics data have been deposited to the ProteomeXchange Consortium via the PRIDE (Vizcaíno et al., 2016) partner repository with the dataset identifier PXD024700.

### Predicting BMCs Shell Proteins

The Hidden Markov Models (HMMs) of two BMC shell protein domains listed as Pf00936 and Pf03319 were retrieved from the Pfam database to predict BMC shell proteins as described in Axen et al., 2014; Zeng et al., 2019. Shell proteins were predicted by a HMM search using the HMMER package and a local protein database of *P. freudenreichii* DSM 20271 genome [CP010341.1 (Deptula et al., 2017)]. All hits with an *e*-value less than or equal to 1e-05 that correspond to a genomic record from Genbank, RefSeq, EMBL, or DDBJ databases were accepted as BMC shell protein homologs (Supplementary File 1).

## Results

### Growth Performance and Metabolite Production on 1,2-Propanediol and L-Lactate

**FIGURE 1:**
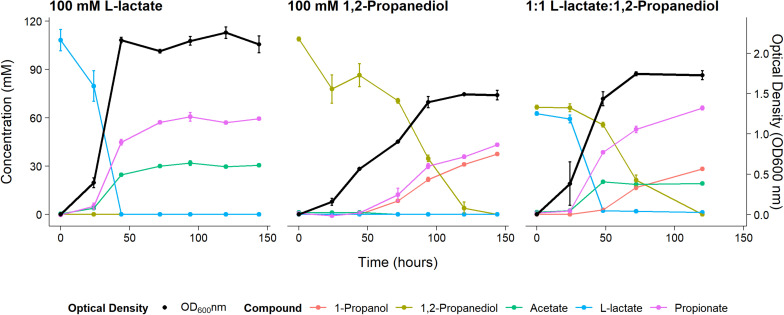
Substrate consumption, metabolite production and biomass formation of *P. freudenreichii* grown on 100 mM L-lactate, 100 mM 1,2-Propanediol, and 1:1 1,2-Propanediol:L-lactate for 6 days. Error bars = standard error (*n* = 3 biological replicates).

Anaerobic growth of *P. freudenreichii* on 100 mM 1,2-propanediol was monitored for 6 days with daily sampling in triplicate. As a control, *P. freudenreichii* was cultured on 100 mM L-lactate. *P. freudenreichii* was able to completely metabolize 100 mM 1,2-propanediol during a period of 6 days (see Figure 1). This resulted in the production of 37.5 ± 0.1 mM 1-propanol and 43.2 ± 0.5 mM propionate. No production of acetate was observed, and with the current HPLC method used, approximately 28 mM of expected C_3_ compounds is missing, conceivably volatile propionaldehyde. In the control cultures, 100 mM L-lactate was consumed within 2 days, resulting in 65.1 + 0.1 mM propionate and 30.5 ± 0.1 mM acetate, close the expected molar ratio of 2:1 (Seeliger et al., 2002). Growth on 1,2-propanediol resulted in slower growth, with a stationary phase after 120 h vs 48 h in L-lactate. Our results clearly demonstrate that *P. freudenreichii* can grow on 1,2-propanediol, metabolizing it to propionate and 1-propanol.

To monitor metabolism of 1,2-propanediol in the presence of other carbon sources *P. freudenreichii* was grown in media containing both L-lactate and 1,2-propanediol. When NAD^+^/NADH pools are shared between the cytosol and the BMC as discussed by Ferlez et al. (2019), the highest ATP yield from 1,2-propanediol in the presence of L-lactate can be obtained by co-fermenting 1,2-propanediol and L-lactate solely to propionate (see Supplementary Text 1). The expected ATP yield with L-lactate and 1,2-propanediol as mixed substrates is higher compared to cells growing solely on L-lactate (see Supplementary Text 1). We found production of 67.1 ± 1.5 mM propionate, 19.0 ± 1.0 mM of acetate, and 28.3 ± 2.8 mM 1-propanol in cells growing on the mixed substrates. Assuming 1-propanol:propionate is produced in a 1:1 ratio from 1,2-propanediol and acetate:propionate is produced in a 1:2 ratio from L-lactate, 28 mM of the propionate originates from the 1,2-propanediol metabolism and 38 mM propionate originates from L-lactate metabolism. This matches the total found propionate of 67.1 ± 1.5 mM propionate in the samples. In mixed substrate conditions the amounts of total products formed indicate no apparent loss of C_3_ compounds, which may indicate reduced loss of volatile propionaldehyde. Biomass formation with 1:1 L-lactate:1,2-propanediol-grown cells was found to be lower compared to 100 mM L-lactate-grown cells. These results indicate no apparent energetic benefit from mixed substrate conditions compared to mono substrate conditions, pointing toward independent pathways. Our results show mixed-substrate metabolism influences the total amount of short-chain fatty acid production, but pathway interactions are not apparent.

### Vitamin B_12_ Production

To demonstrate active vitamin B_12_ production under 1,2-propanediol utilizing conditions, *P. freudenreichii* was grown for 7 days in either yeast extract medium supplemented with 66 mM L-lactate or 49 mM 1,2-propanediol. Biomass formation, substrate utilization, and vitamin B_12_ production by *P. freudenreichii* was monitored after 7 days of incubation. Incubation vessels were not opened in-between to prevent side effects by oxygen-dependent stimulation of vitamin B_12_ production, as has been shown for *P. freudenreichii* (Quesada-Chanto et al., 1998). Again, complete utilization of L-lactate and 1,2-propanediol was found (Supplementary File 2).

**FIGURE 2:**
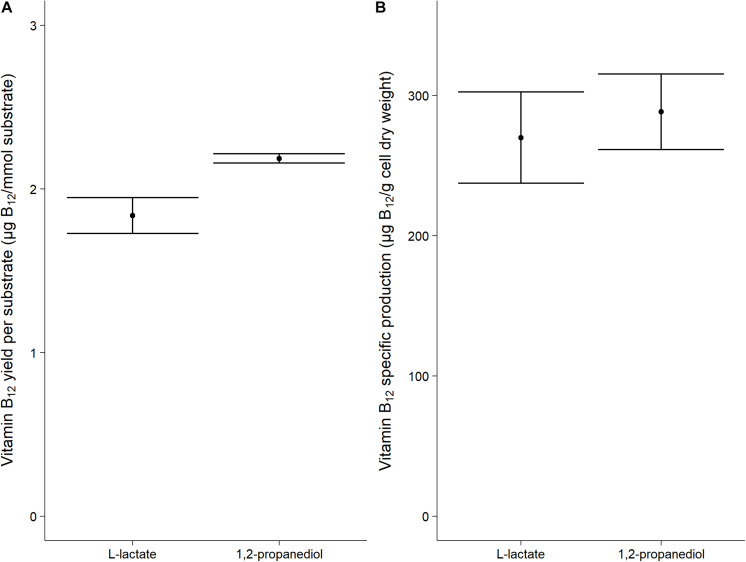
Vitamin B_12_ formation in cells grown on L-lactate or 1,2-propanediol. **(A)** vitamin B_12_ yield in μg/mmol substrate **(B)** Biomass specific vitamin B_12_ production in μg/g cell dry weight. Error bars = standard error (*n* = 3 biological replicates).

Vitamin B_12_ was produced both when grown on L-lactate as on 1,2-propanediol (1.84 ± 0.11 μg/mmol L-lactate vs 2.19 ± 0.03 μg/mmol 1,2-propanediol). The specific vitamin B_12_ production when grown on L-lactate and on 1,2-propanediol was 270 ± 33 μg/g cells and 288 ± 27 μg/g cells, respectively, (Figure 2). No significant difference was thus found for the production of vitamin B_12_ per g cells when growing on either L-lactate supplemented media or 1,2-propanediol supplemented media. In complex media, 1,2-propanediol as carbon source thus supports vitamin B_12_ production by *P. freudenreichii*.

### Proteomics and Electron Microscopy of L-Lactate and 1,2-Propanediol Grown Cells

**FIGURE 3:**
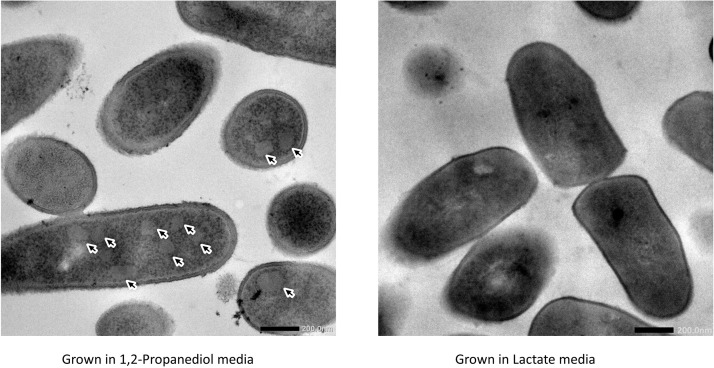
Visualization of bacterial microcompartments in propanediol media **(left)** and absence in lactate media **(right)**.

Cells growing in media supplemented with L-lactate (control) and with 1,2-propanediol were visualized using TEM. Thin sections of cells grown on 1,2-propanediol supplemented media clearly display cellular structures which were not found in the cells grown on L-lactate supplemented media (see Figure 3), and those structures resemble BMC structures found in other bacteria including *L. monocytogenes* (Zeng et al., 2019), *S. enterica* (Crowley et al., 2008), and *E. coli* (Toraya et al., 1979).

**FIGURE 4:**
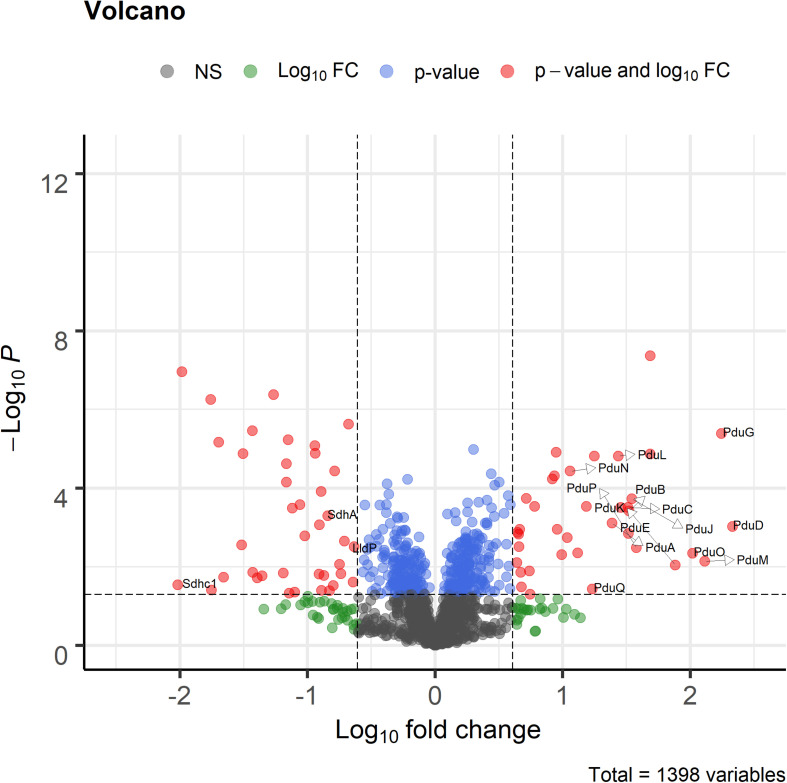
Volcano plot of proteomic analysis of cells grown in 1,2-propanediol media and L-lactate media. Positive log_10_ fold change indicate upregulation in 1,2-propanediol-grown cells, negative log_10_ fold change indicate down regulation in 1,2-propanediol-grown cells compared to lactate-grown cells. Red dots indicate proteins with *p* < 0.05 and 4-fold difference expression. Blue dots indicate only *p* < 0.05, green dots only 4-fold difference. Black dots indicate non-significant, non-differentially expressed proteins (*n* = 3 biological replicates).

Proteome analysis revealed that BMC structural shell proteins (PduA, PduB, PduK, PduJ, PduN, and PduM) and enzymes involved in 1,2-propanediol utilization (PduL, PduC, PduD, PduE, PduP, PduO, and PduQ) were significantly more abundant in cells grown with 1,2-propanediol as a substrate compared to cells grown on L-lactate (Figure 4). Lactate permease, succinate dehydrogenase subunits A, C1 (SdhA, SdhC1) were found to be significantly more abundant in the L-lactate-grown cells compared to 1,2-propanediol-grown cells, indicating a decrease of proteins in lactate-degradation pathways such as the Wood-Werkman cycle. Gene set enrichment analysis of KEGG pathways (see Supplementary File 3) revealed upregulation of the propanediol degradation pathway in 1,2-propanediol grown cells compared to L-lactate grown cells (adjusted *p*-value < 0.10). DNA repair mechanisms were also found to be activated in 1,2-propanediol-grown cells (adjusted *p*-value < 0.10). In 1,2-propanediol-grown cells significant suppression of oxidative phosphorylation was found compared to L-lactate grown cells (adjusted *p*-value < 0.01). The measured consumption of 1,2-propanediol, production of 1-propanol and propionate, visualized cellular structures, and strong upregulation of the *pdu* loci proteins provide evidence for activation of BMC-dependent 1,2-*pdu* in *P. freudenreichii* DSM 20271.

### Overview of BMC-Dependent 1,2-Propanediol Metabolism Model in *P. freudenreichii*

**FIGURE 5:**
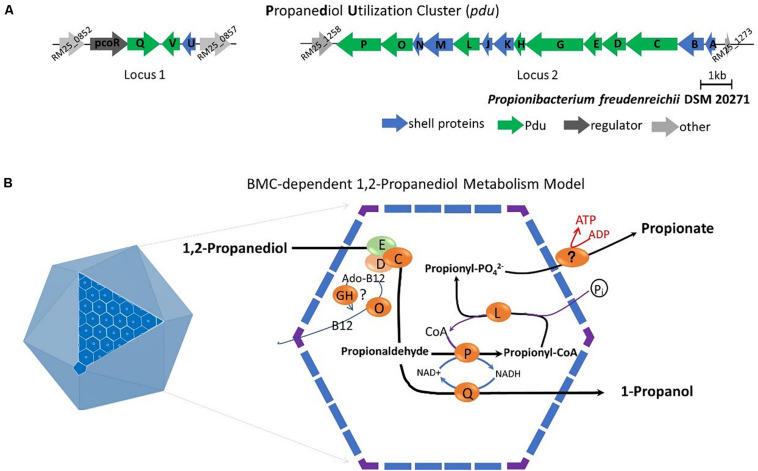
**(A)** Analysis of the *pdu* gene cluster (Details in Supplementary File 1). Characters in orange represent Pdu enzymes, in blue represent BMC shell proteins, in dark represent regulator and in gray represent unannotated proteins with gene ID. **(B)** Model of BMC-dependent 1,2-propanediol metabolism. In the left, the icosahedral diagram represent BMC with one surface showing the assembly of shell proteins. In the right, the metabolic pathway of 1,2-propanediol metabolism. PduCDE, B_12_-dependent diol dehydratase; PduP, CoA-dependent propionaldehyde dehydrogenase; PduGH, diol dehydratase reactivase; PduO, corrinoid adenosyltransferase; PduS, Vitamin B_12_ reductase; PduL, phosphate propanoyltransferase; PduW, propionate kinase; and PduQ, propanol dehydrogenase. See text for details.

Subsequent analysis of the *pdu* cluster in *P. freudenreichii* DSM 20271 identified two distant loci, with locus 1 starting from RM25_0852 to RM25_0857 and locus 2 starting from RM25_1258 to RM25_1273 (Figure 5A). Locus 1 contains four genes which are *pocR* encoding a transcriptional regulator, *pduQ* encoding 1-propanol dehydrogenase, *pduV* with unknown function and *pduU* encoding BMC shell protein. Locus 2 carries 14 genes including 6 genes encoding BMC shell proteins and 8 genes encoding enzymes for the 1,2-propanediol degradation pathway and is not preceded by any known transcriptional regulators.

Based on the structural studies of BMCs (Sutter et al., 2017; Greening and Lithgow, 2020) and our understanding of 1,2-propanediol metabolism (Sampson and Bobik, 2008; Kerfeld et al., 2018; Zeng et al., 2019), we propose the model of BMC-dependent 1,2-propanediol metabolism in *P. freudenreichii*, with the predicted BMC shell proteins PduA, PduB, PduK, PduJ, PduM, PduN, and PduU constituting the self-organized icosahedral organelle (Kerfeld et al., 2018; Greening and Lithgow, 2020). As illustrated in Figure 5B, the catabolism of 1,2-propanediol starts with the conversion of 1,2-propanediol to propionaldehyde by vitamin B_12_-dependent diol dehydratase PduCDE. The toxic propionaldehyde is then converted to propionate by the enzyme CoA-dependent propionaldehyde dehydrogenase PduP, followed by action of phosphate propanoyltransferase PduL, and potential propionate kinase located in the cytoplasm, resulting in the end product propionate and the production of ATP. The other end product is produced following conversion of propionaldehyde by propanol dehydrogenase PduQ into 1-propanol. The diol dehydratase reactivase PduGH and corrinoid adenosyltransferase PduO are linked to the supply and recycling of vitamin B_12_.

## Discussion

*Propionibacterium freudenreichii* is commonly found in the rumen and colon of animals and in the human intestine (Bryant, 1959). In these environments fucose and rhamnose are degraded to 1,2-propanediol by the present microbiota (Xue et al., 2008). We showed 1,2-propanediol can be further metabolized into propionate and 1-propanol by *P. freudenreichii*, thereby supporting anaerobic growth. Previously, a locus containing 15 genes involved in *pdu* was detected in *P. freudenreichii* (Falentin et al., 2010). *In vivo* gene expression analysis showed the *pdu* operon to be expressed in *P. freudenreichii* cells contained in the colon environment of a pig (Saraoui et al., 2013), pointing toward *pdu* in intestinal environments. Interestingly, in this study we identified the *pdu* cluster distributed in two different loci in *P. freudenreichii* DSM 20271. The presence of the *pdu* cluster seems to be species specific in propionic acid bacteria, as blasting the *pdu* cluster of *P. freudenreichii* against *Cutibacterium acnes, Acidipropionibacterium acidipropionici, A. thoenii*, and *A. jensenii* did not result significant hits for key components in the cluster (see Supplementary File 4). Presence of 1,2-propanediol in the medium induced expression of the two loci in *P. freudenreichii* DSM 20271 and resulted in BMC formation, 1,2-propanediol metabolism and consequently propionate and 1-propanol production. Our results show that expression of the *pdu* cluster from two different loci results in effective BMC formation and 1,2-propanediol metabolism. Indeed, BMC genes are split into two or more loci in 40% of the prokaryotic genomes containing BMCs (Abdul-Rahman et al., 2013). In *P. freudenreichii* DSM 20271, locus 1 is preceded by transcriptional activator *pocR*, which has been linked to activation of the *pdu* cluster in *Salmonella* (Chen et al., 1994). We did not find any annotated transcriptional regulator in the vicinity of locus 2. The presence of two different loci in many prokaryotic genomes suggests expression is controlled by additional regulators next to PocR. Indeed heterologous expression of BMCs uncoupled from their cognate transcriptional regulators has been reported previously (Wilson, 2021). The transcriptional regulation and activation of the two *pdu* loci and the role of *pocR* in *P. freudenreichii* requires further attention.

Upregulation of the *pdu* cluster and DNA repair mechanisms clearly indicated the crucial role of BMCs to protect *P. freudenreichii* from the toxic intermediate propionaldehyde produced in the degradation pathway. The ability to utilize substrates producing toxic intermediates upon degradation results in a competitive advantage to other gut microbiota (Jakobson and Tullman-Ercek, 2016), as shown for ethanolamine utilization by *S. enterica* during intestinal inflammation (Thiennimitr et al., 2011). Interestingly, the presence of genes encoding metabolosomes for the utilization of ethanolamine and propanediol has been linked to pathogenicity and aids in anaerobic growth and colonization of foodborne pathogens *L. monocytogenes, C. perfringens*, and *S. typhimurium* (Korbel et al., 2005; De Weirdt et al., 2012). It has been suggested for beneficial bacterium *L. reuteri* that competition for 1,2-propanediol could result in decreased proliferation of pathogens (Cheng et al., 2020). *P. freudenreichii* is considered to be non-pathogenic and has the generally recognized as safe (GRAS) status (Meile et al., 2008). Substrate competition for 1,2-propanediol by *P. freudenreichii* may thus exert similar effects as suggested for *L. reuteri* on growth of pathogenic bacteria. This is in line with reports that *P. freudenreichii* can decrease adhesion of pathogens to human intestinal mucus cells (Collado et al., 2007). Furthermore, *P. freudenreichii* stimulates the growth of beneficial bifidobacteria (Satomi et al., 1999; Hojo et al., 2002) thereby promoting a healthy gut microbiota. The role of BMC-mediated 1,2-propanediol utilization by *P. freudenreichii* and its importance for modulating gut microbiota composition, both by substrate competition and promoting other beneficial microbiota, requires further investigation.

Our study shows the potential of *P. freudenreichii* to substantially contribute to the production of propionate in the human gut. Next to 1,2-propanediol, also lactic acid (Macfarlane and Gibson, 1997) is a major end-product of microbial fermentation, which consequently can also be fermented by *P. freudenreichii* and additionally contributes to the production of propionate. In mixed substrate conditions both pathways remained active, albeit without apparent interaction based on metabolite formation. However, mixed substrate conditions decreased the loss of C_3_, suggesting less loss of volatile propionaldehyde, thus potentially more efficient BMC assembly. The assembly of BMCs in mixed substrate condition requires further attention. Propionate is linked to many putative health effects [reviewed by (Hosseini et al., 2011)] and can further stimulate bifidobacteria (Kaneko et al., 1994). The production of propionate from 1,2-propanediol further supports the potential of *P. freudenreichii* as a probiotic.

The metabolism of 1,2-propanediol requires propanediol dehydratase, which is vitamin B_12_-dependant (Axen et al., 2014). Vitamin B_12_ is actively produced by *P. freudenreichii* as it is essential cofactor in a key enzyme in the Wood-Werkman cycle, methylmalonyl-CoA mutase. Synthesis of vitamin B_12_ in *P. freudenreichii* follows the anaerobic pathway (Roessner et al., 2002), enabling production of vitamin B_12_ for anaerobic metabolism of lactate. Here we show vitamin B_12_ is produced in similar amounts in complex medium when cells metabolize 1,2-propanediol or L-lactate as carbon source. In *S. typhimurium pocR* mediated expression of vitamin B_12_ is induced in the presence of propanediol (Richter-Dahlfors et al., 1994). We also identified *pocR* upstream of *pdu* loci 1 in *P. freudenreichii* DSM 20271, but as discussed before its exact regulatory role in *P. freudenreichii* remains to be elucidated. Vitamin B_12_ production is also regulated by a vitamin B_12_ regulated riboswitch in *P. freudenreichii.* In *Propionibacterium* strain UT1 expression of vitamin B_12_ biosynthesis occurred at vitamin B_12_ concentrations of 750 μM, much higher compared to the vitamin B_12_ concentrations found in this study (∼90 nM). The role of this riboswitch for vitamin B_12_ production during metabolism of 1,2-propanediol also remains to be elucidated. Based on our findings, we hypothesize that *P. freudenreichii* occupies a lactate and propanediol-rich niche in the gut environment. Symbiotic relationships have been shown for the production of vitamin B_12_ (Belzer et al., 2017; Sokolovskaya et al., 2020), which may be hard-wired in the vitamin B_12_ production of *P. freudenreichii*. As next to vitamin B_12_-dependent lactate metabolism, BMC-mediated 1,2-propanediol metabolism supports anaerobic growth of *P. freudenreichii*, thereby contributing to *in situ* vitamin B_12_ production in the gut.

This study presents evidence for BMC-mediated vitamin B_12_-dependent utilization of 1,2-propanediol in *P. freudenreichii.* We have shown that 1,2-propanediol supports anaerobic growth of *P. freudenreichii*. It is conceivable that utilization of 1,2-propanediol could aid colonization of *Propionibacterium freudenreichii* in the human gut to exert beneficial effects, such as delivering vitamin B_12_ and propionate *in situ*, but these aspects require further study.

## Data Availability Statement

The original contributions presented in the study are publicly available. This data can be found here: https://www.ebi.ac.uk/pride/archive/projects/PXD024700.

## Author Contributions

AD and ZZ designed and performed the experiments. SB, AD, and ZZ performed proteomics and analyzed data. ZZ, AD, ES, RN, and TA analyzed data. ZZ, AD, ES, and TA wrote the manuscript. All authors read, edited and approved the manuscript.

## Conflict of Interest

The authors declare that the research was conducted in the absence of any commercial or financial relationships that could be construed as a potential conflict of interest.
