# Supplementary material for: Bacterial Microcompartment-Dependent 1,2-Propanediol Utilization of Propionibacterium freudenreichii
Source: Front Microbiol. 2021 May 12;12:679827. doi: 10.3389/fmicb.2021.679827 (PMC8149966; doi:10.3389/fmicb.2021.679827)
Supplement: Supplementary file 3 [file Data_Sheet_3.DOCX]

**Supplementary text 1: Energetics with pathway interactions between bacterial microcompartment (BMC) and Wood-Werkman cycle**

The BMC mediated degradation of 1,2-propanediol to propionyl-phosphate and propionate generates one NADH and one ATP. Degradation of lactate to propionate through the Wood-Werkman cycle consumes one NADH and by the anaerobic electron transport from lactate to fumarate translocates 2 H^+^, thereby generating 0.5-0.67 ATP. This means that if lactate and PD are co-metabolised in a 1:1 ratio, the yield obtainable by fermentation of 50 mM Lactate to propionate is 33 mM ATP by fumarate respiration and the total yield of fermentation of 50mM 1,2-Propanediol to propionate is 50 mM ATP by substrate-level phosphorylation. A total yield of 83 mM ATP and sole production of 100 mM propionate would be expected if NAD^+^/NADH pools are shared. If co-factors cannot be shared due to the limitations by shell proteins, the general accepted pathways for both lactate and 1,2-propanediol are expected. Fermentation of 50 mM lactate would result in production of 33 mM propionate + 17 mM acetate, yielding 39 mM ATP (17 mM by substrate level phosphorylation of acetate and 22 mM by fumarate reduction producing propionate). Fermentation of 50 mM PD would result in production of 25 mM propionate and 25 mM 1-propanol, yielding 25 mM ATP. A total production of 58 mM propionate + 17 mM acetate + 25 mM 1-propanol would be expected, yielding a total 64 mM ATP. Hence, sharing NAD^+^/NADH pools would be energetically beneficial. Fermentation of 100 mM Lactate results in 78 mM ATP and fermentation of 100 mM 1,2-PD results in 50 mM ATP. Hence, the biomass formation for 50mM PD:50 mM lactate grown-cells using a shared co-factor pool is expected to be higher compared to 100 mM L-lactate grown-cells, whereas if the co-factor pool is not shared the expected biomass formation is lower compared to 100 mM L-lactate grown cells, but higher compared to 100 mM 1,2-PD grown-cells
