# Supplementary material for: Bacterial Microcompartment-Dependent 1,2-Propanediol Utilization of Propionibacterium freudenreichii
Source: Front Microbiol. 2021 May 12;12:679827. doi: 10.3389/fmicb.2021.679827 (PMC8149966; doi:10.3389/fmicb.2021.679827)
Supplement: Supplementary file 5 [file Table_1.DOCX]

| **Sample** | **mMol substrate utilized** | **Theoretical yield ATP per mol substrate** | **Biomass yield (g CDW/L)** |
| --- | --- | --- | --- |
| L-lactate | 65.8 | 0.78 | 0.45 |
| 1,2-Propanediol | 49.1 | 0.50 | 0.38 |

Supplementary file 2: Substrate consumption and biomass production for cells grown for B_12_ assay
